# Trends in clinical trial investigator workforce and turnover: An analysis of the U.S. FDA 1572 BMIS database

**DOI:** 10.1016/j.conctc.2019.100380

**Published:** 2019-05-21

**Authors:** Christopher B. Fordyce, Matthew T. Roe, Christine Pierre, Terri Hinkley, Gerrit Hamre, Pamela Tenaerts, Jonathan McCall, James D. Topping

**Affiliations:** a Duke Clinical Research Institute, Durham, NC, USA; b Division of Cardiology, University of British Columbia, Vancouver, British Columbia, Canada; c Society for Clinical Research Sites, Ellicott City, MD, USA; d Academy of Medical-Surgical Nurses, Sewell, NJ, USA; e Clinical Trials Transformation Initiative, Durham, NC, USA

**Keywords:** Investigator turnover, Drug trials, FDA

## Abstract

**Background:**

High turnover rates among clinical trial investigators contribute to inefficiency, instability, and increased costs for the clinical research enterprise; however, factors contributing to investigator turnover have not been well characterized.

**Methods:**

Using information from the U.S. Food and Drug Administration's Bioresearch Monitoring Information System (BMIS), we examined trends in the overall clinical investigator workforce and within specific “phenotypes” as well as differences by investigator location (U.S.-based vs. non-U.S.-based). We identified unique investigators within the database, stratifying them into one of three “phenotypes”: those with one Form FDA1572 submission across the study interval (“one-and-done”); those with two or more submissions but with substantial intervals between trials (“stop-and-go”); and those with two or more submissions and continuous involvement in multiple trials (“stayers”).

**Results:**

Of the 172,453 unique investigators who submitted a Form FDA 1572 during the study interval (1999–2015), 85,455 were classified as “one-and-done” investigators; 21,768 as “stop-and-go” investigators; and 65,231 as “stayer” investigators. The total number of investigators declined across the study interval. Among all subgroups, only “one-and-done” investigators showed growth across the study period, largely driven by increases in non-U.S.-based investigators. “Stop-and-go” investigators showed declines for both U.S.-based and non-U.S.-based investigators, as did “stayers,” who showed the largest absolute and proportional declines of all subgroups.

**Conclusions:**

From 1999 to 2015, investigators submitting a Form FDA 1572 to the BMIS database declined by approximately one-third and the proportion of investigators involved in only one trial increased, signaling potential adverse trends in the clinical investigator workforce. Strategies for sustaining investigator engagement warrant further exploration.

## Highlights

Clinical Significance.

• High turnover rates among clinical trial investigators contribute to inefficiency, instability, and increased costs for the clinical research enterprise.
• Using data from the FDA Bioresearch Monitoring Information System (BMIS), we identified 172,453 unique investigators who submitted a Form FDA 1572 during the study interval (1999–2015). Of these, 85,455 were classified as having done only a single study (“one-and-done” investigators); 21,768 as being intermittently involved in research activity (“stop-and-go” investigators); and 65,231 as investigators who were continuously involved in clinical research (“stayer” investigators).
• From 1999 to 2015, investigators submitting a Form FDA 1572 to the BMIS database declined by approximately one-third and the proportion of investigators involved in only one trial increased, signaling potential adverse trends in the clinical investigator workforce.

## 1 Introduction

Concerns have emerged in some sectors of the clinical trials enterprise regarding an evident stagnation in drug development and mounting difficulties in achieving marketing approval for candidate therapies [1] despite a high global disease burden [2]. The failure rate along the entire T1 spectrum has remained stubbornly high over decades and is estimated to be nearly 90% from candidate molecule to marketing approval [3]. Multiple factors are likely responsible for these challenges, including the globalization of clinical research [[4], [5], [6], [7]] and steep increases in the costs of bringing new therapies to market [1]. Further, as Califf and Harrington note, there is limited evidence that the U.S. clinical trials system produces data that are of higher quality or better managed than data collected outside the United States [8].

However, another factor that may deserve closer scrutiny is the persistent concern among the clinical research community about high rates of turnover for U.S. clinical investigators and a declining U.S. clinical trials workforce as a whole [[9], [10], [11], [12], [13], [14], [15]], both of which are believed to contribute to inefficiency, instability, and increased costs for clinical trials [12,13]. A survey published in 2015 by the Tufts Center for the Study of Drug Development found that as of 2013, approximately half of all clinical investigators worldwide had filed a Form FDA 1572 for the first time, and that rates of turnover among experienced investigators appeared to be worsening [13]. In addition, rates of growth in the overall number of new investigators worldwide appeared to be slowing, and the proportion of North American investigators has declined relative to the overall pool of investigators [13]. The Tufts study also found that although rates of turnover were highest among the least active investigators, they were also increasing among more experienced investigators [13].

Sponsors, funding agencies, academic institutions, and other stakeholders invest substantial time and resources to initiate new investigators into the conduct of clinical trials and to ensure that established investigators remain active in clinical research. But despite concerns about investigator turnover, relatively few empiric data exist to help quantify the current landscape of clinical investigator turnover in the United States [13,16], and precise characterization of the national and international clinical investigator workforce has proven elusive [15]. Further, to our knowledge, there are no data that use patterns of engagement in the research enterprise to describe multiple “phenotypes” of investigators, including those who perform only one clinical trial and then leave site-based research versus those who remain continuously engaged in clinical research.

In this exploratory observational analysis, we sought to: 1) describe trends in the overall clinical trial investigator workforce; 2) delineate trends within specific investigator phenotypes, including investigators involved in only one trial (“one-and-done”), investigators involved in multiple trials but with substantial intervals between trials (“stop-and-go”), and investigators continuously involved in multiple trials (“stayers”); and 3) determine potential differences in trends according to location of investigator (U.S.-based vs. non U.S.-based).

## 2 Methods

The Clinical Trials Transformation Initiative (CTTI) (http://www.ctti-clinicaltrials.org) is a public-private partnership co-founded by the U.S. Food and Drug Administration (FDA) and Duke University that seeks to identify and drive adoption of practices that increase the quality and efficiency of clinical trials. The research methods described below are part of a larger project supported by CTTI on strengthening the investigator site community [17].

### 2.1 Data acquisition

Sponsors who conduct drug trials under regulations pertaining to Investigational New Drug applications are asked to submit a completed FDA Statement of Investigator form (Form FDA 1572) for each clinical investigator. This form documents the clinical investigator's qualifications and agreement to comply with FDA regulations as well as information about the clinical trial site. Investigators are subsequently listed in the Bioresearch Monitoring Information System (BMIS) database [19] if sponsors complete the optional step of submitting the form to the FDA. The BMIS database, which is publicly available and can be downloaded from the FDA website, served as the primary source of data for our analysis. Importantly, because 1) submission of a Form FDA 1572 is voluntary and 2) because sponsors may conduct clinical trials outside of the United States without an IND application, the BMIS database may not reflect the entire scope of investigator activity.

We downloaded a version of the FDA 1572 BMIS database on Nov 14, 2016 (http://www.accessdata.fda.gov/scripts/cder/bmis/). Subsequent analyses were performed by an experienced clinical research informaticist (J.T.). Data were loaded and analyzed using STATA 12.1 (StataCorp LLC, College Station, TX). We included all database entries from 1999 to 2015, inclusive. Entries were excluded if the last name was “??” or null. The final dataset included 746,641 entries.

### 2.2 Investigator phenotypes

Unique investigators were identified using a unique last and first name combination, generating 172,453 investigators. In our preliminary work with these data, we found that database information entered for the same investigator often differed slightly, so that adding additional variables from the database to increase specificity would often have the effect of separating an investigator's entries from one another. We opted to use solely the last and first name combination even though it meant that data for some frequently occurring names would be collapsed together. Each unique name combination was assigned an ID, and subsequent Form FDA 1572 submissions within that ID were ordered. We assumed that both the number and frequency of entries by an investigator would best reflect the level of research activity. To this end, investigators were further stratified into three “phenotypes” according to patterns of Form FDA 1572 application submission, as described below:

**“One-and-Done” Investigators.** This category describes investigators with only one Form FDA 1572 submission across the entire study interval. These investigators are no longer actively involved in leading FDA-regulated drug trials.

**“Stop-and-Go” Investigators.** Investigators characterized as “stop-and-go” had at least two Form FDA 1572 submissions, but with the time to second submission occurring beyond the 75th percentile of the interval between the first and second submissions. These are investigators who have remained active in research but are relatively less experienced than the “stayer” population described below, based on the interval between the first and second submissions.

**“Stayer” Investigators.** Stayer investigators were those with at least two Form FDA 1572 submissions, but with the time to second submission occurring within the 75th percentile of the interval between the first and second submissions. These are investigators who have remained active in leading FDA-regulated drug trials on a relatively continuous basis. Based on the interval between first submission and second submission, we considered these investigators to be the most experienced of the three groups.

Each unique ID fell into one of these categories, and IDs were counted once. Subsequent submissions (third and beyond) were not considered. Notably, these data reflect the number of site principal investigators, and not the pool of sub-investigators.

### 2.3 Determining the interval between first and second submission

Several variables from the BMIS database were considered as part of determining the interval between first and second submission of Form FDA 1572 (Table 1). The three investigator subgroups were further separated by location, determined by whether the country field indicated “USA” or another value (Supplemental Fig. 1).

**Table 1:** Variables considered for determining the interval between submission of Form FDA 1572 to BMIS database.

Variable	Description
Newid	Assigned based on a unique first and last name combination
Obs	Data were ordered by receipt date and assigned a sequential number
Firsub	This is the minimum receipt date for a newid- their first submission receipt date and the date on which an investigator is plotted
Secdiff	This is the difference between the receipt date and Firsub for observation 2; i.e., number of days until the second submission
Oad	This is a flag. 1 is assigned if the maximum observations for a newid = 1

### 2.4 Choice of 75th percentile cutoff

The 75th percentile was chosen empirically and based on a value of 1013 days, which was felt to be a reasonable interval for distinguishing more experienced versus less experienced researchers among those submitting more than one Form FDA 1572 application. The 50^th^ percentile (411 days) was also considered; however, the study team felt that there would be investigators phenotypically classed as “stayers” working on larger clinical trials who would not have necessarily completed a Form FDA 1572 within this interval and thus would be underrepresented.

## 3 Results

**Fig. 1:**
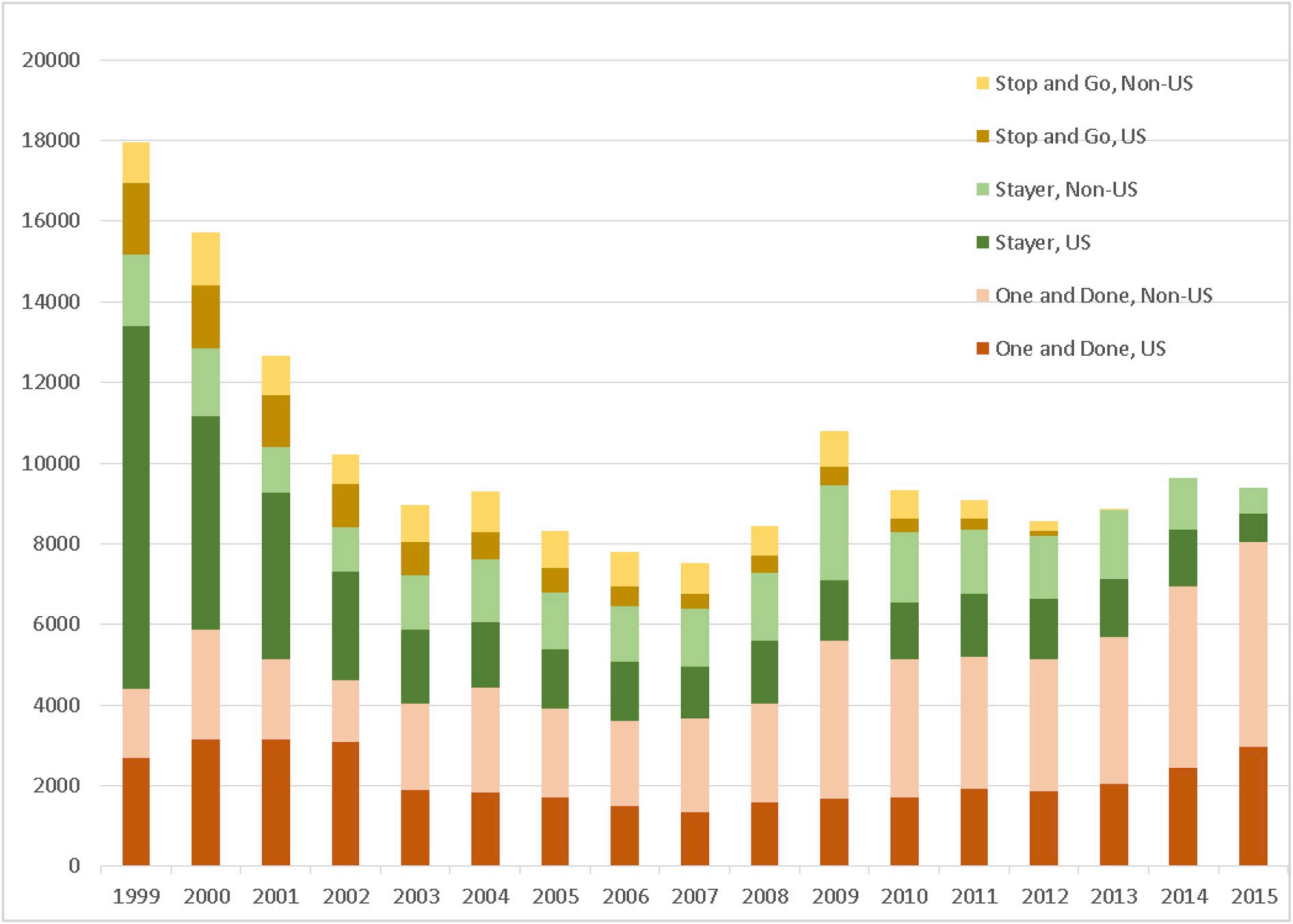
Temporal trends in investigator turnover, all investigators, 1999–2015.

Our examination of data contained in the FDA BMIS database showed a total of 172,453 unique clinical trials investigators who had submitted a Form FDA 1572 during the study interval of 1999–2015. Of these, 85,455 (49.6%) were classified as “one-and-done” investigators; 21,768 (12.6%) were classified as “stop-and-go” investigators; and 65,231 (37.8%) were classified as “stayer” investigators. The total number of investigators conducting any FDA-regulated drug trials showed an overall decline from 17,941 in 1999 to 9387 in 2015. However, this decline was not uniformly continuous from year to year, with an absolute nadir for the study period observed in 2007 (n = 7509) (Fig. 1).

We also observed several distinct temporal trends across the different investigator subgroups. Out of all subgroups, only the category of “one-and-done” investigators showed growth across the study period, a change largely driven by increases in the number and proportion of non-U.S.-based “one-and-done” investigators (Fig. 2a; Supplemental Fig. 2a).

**Fig. 2a:**
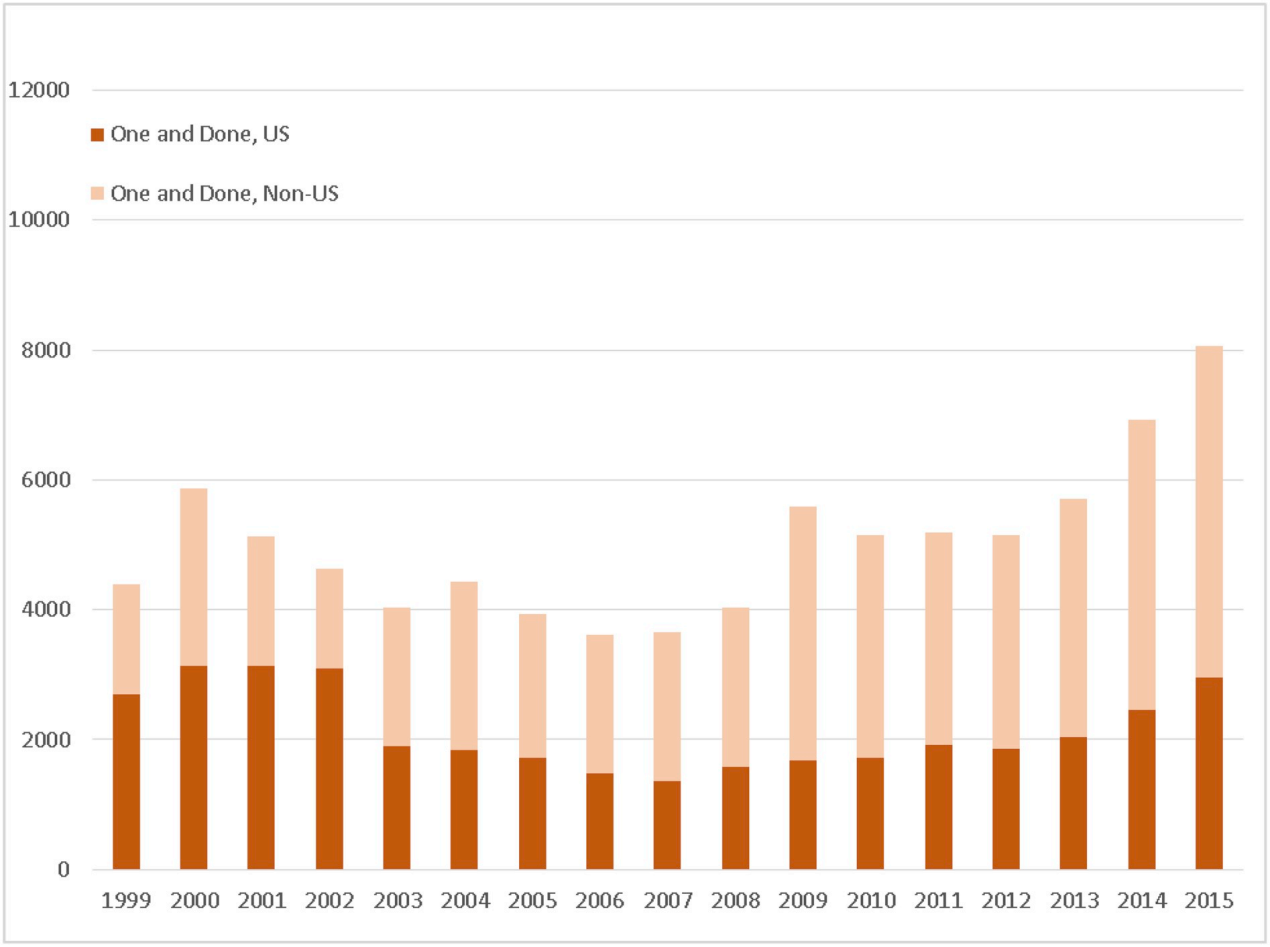
Temporal trends in investigator turnover, 1999–2015, according to subgroup “one-and-done.” Investigators were classed as one-and-done if they had only 1 Form FDA 1572 submission across the entire study interval.

The “stop-and-go” subgroup showed relatively consistent declines across the study interval for both U.S. and non-U.S. investigators (Fig. 2b; Supplemental Fig. 2b), as did the “stayer” subgroup, which had the largest declines of all subgroups, both in total numbers of investigators and as a proportion of investigators as a whole (Fig. 2c; Supplemental Fig. 2c).

**Fig. 2b:**
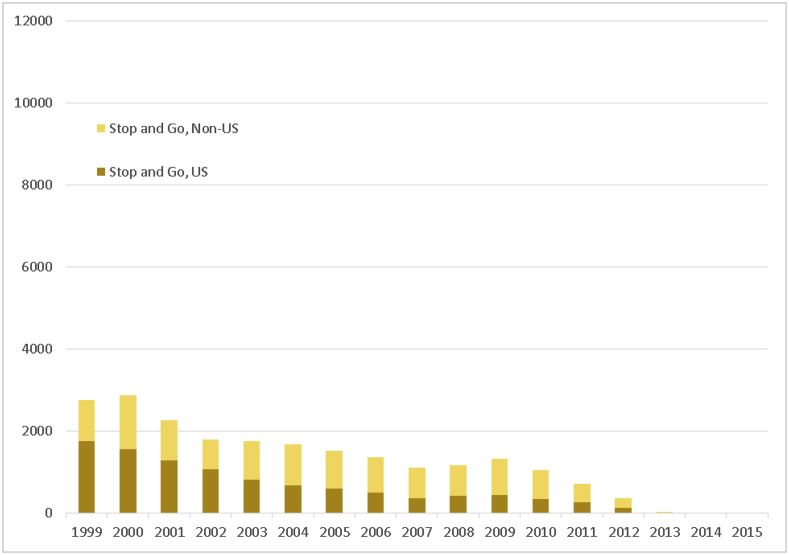
Temporal trends in investigator turnover, 1999–2015, according to subgroup “stop-and-go.” Investigators were classed as stop-and-go if they had at least 2 Form FDA 1572 submissions, but with the time to second submission occurring beyond the 75th percentile of the interval between the first and second submissions. Note: There were very few “stop-and-go” investigators after 2012 due to the 1013-day minimum period between first and second submissions required to be classified in this category.

**Fig. 2c:**
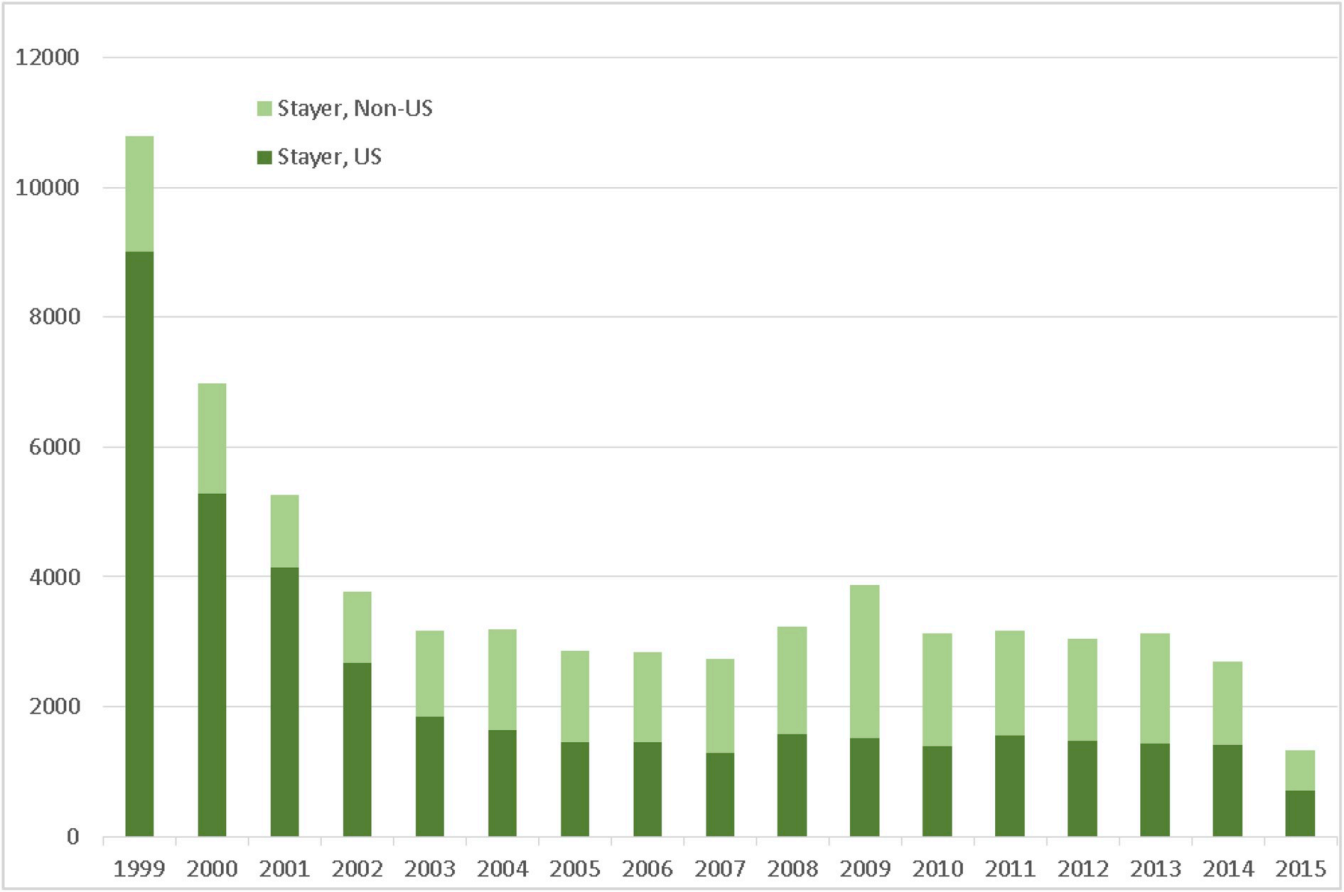
Temporal trends in investigator turnover, 1999–2015, according to subgroup “stayer.” Investigators were classed as stayers if they had at least 2 Form FDA 1572 submissions, but with the time to second submission occurring within the 75th percentile of the interval between the first and second submissions.

Notably, the “stayer” subgroup also showed the most pronounced change in the proportions of U.S. versus non-U.S. investigators, with the former comprising a substantial majority of all investigators in 1999 (9003 [83.4%] vs. 1787 [16.6%]) but falling to near-parity with non-U.S.-based investigators by 2015 (697 [52.4%] vs. 633 [47.6%]).

Examination of investigator subgroups by location showed similar patterns. Among all subgroups, only the “one-and-done” group showed consistent growth in the number of investigators over time, and then only among non-U.S. investigators (although there was an evident temporal trend toward consistent growth in the number of U.S. “one-and-done” investigators from a low in 2007–2015, this growth did not match high points seen from 2000 to 2002 (Fig. 2a). However, both “stop-and-go” (Fig. 2b) and “stayer” groups (Fig. 2c) showed consistent declines in the numbers of both U.S. and non-U.S.-based investigators, although the latter tended to increase as a proportion of all investigators in both “stop-and-go” and “stayer” subgroups.

## 4 Discussion

Our study describes temporal changes in the overall U.S. clinical trial investigator workforce, including changes in specific investigator phenotypes over time, as reflected in voluntarily reported data available to us in the FDA's BMIS database. We also characterized temporal trends according to investigator location (U.S.-based vs. non-U.S.-based). We found that from 1999 to 2015 the number of clinical trial investigators submitting a Form FDA 1572 declined by approximately one-third. Further, we found that over time, investigators participating in FDA-regulated drug trials were more likely to have participated in only a single clinical trial (“one-and-done” investigator) rather than multiple trials (“stop-and-go” and “stayer” investigators). Finally, we observed a temporal shift from predominately U.S.-based investigators to non-U.S.-based investigators across all three research participation subgroups.

Our results extend previous observations regarding the potential non-sustainability of U.S.-based investigator pool and site-based research [13,15]. As noted previously, a 2015 analysis from the Tufts Center for the Study of Drug Development found reduced rates of growth in the global pool of clinical investigators, with an annual growth rate of 3.3% (for 2013) versus 4.1% over the prior 4 years and an average growth rate of 5.6% over the last 15 years [13]. Although limitations of our data source preclude definitive conclusions, our findings suggest the possibility that there has been little or no further growth in the overall investigator workforce. We also found that compared to 15 years ago, the majority of current investigators are “one-and-done,” and predominately non-U.S. based.

There is longstanding recognition of the need to maintain a robust clinical research infrastructure capable of supporting the trials needed to bring innovative, effective, and safe therapeutics to patients [[20], [21], [22]]. However, it is concerning that our analysis underscores other findings suggestive of a dwindling pool of experienced U.S. investigators [11,13,15]. Further, recent data published by Viergever et al. show that international clinical trial registration, including through ClinicalTrials.gov, has increased by at least sevenfold from 2004 to 2013, although at least some of this increase is presumably due to additional national, international, and organizational requirements for registering studies going into effect [23]. This discrepancy between the investigator pool and clinical trial activity has the potential to profoundly impede novel drug development and the ability to provide optimal care for patients who may putatively benefit from these therapies.

Although some previous studies have used similar approaches to characterizing differential participation by clinical researchers [13,15], our empirical approach to creating objectively defined phenotypes for a “stayer” population versus a “stop-and-go” population based on the interval between filings of Form FDA 1572 represents an innovative bioinformatics approach that allows more granular characterization of the population of investigators, subject to the fundamental limitations of the database. Previous studies have identified only one-time 1572 investigators based on whether multiple filings were performed, and thus were unable to further characterize those investigators with multiple filings [13,24,25]. Our methods allowed us to more accurately identify the group of investigators who remain involved in FDA-regulated drug trials, show more definitively that this phenotype is becoming less common over time, and provide future opportunities to reach out to individual investigators to understand reasons why they remain in clinical research.

Many documented barriers have been associated with a reluctance to continue in research [11,12,15,26], and our recent related study underscores this point among a cohort definitively identified as comprising “one-and-done” investigators [18]. One particularly salient finding from this investigation was that among this group of researchers, nearly half (45%) expressed an interest in continuing to participate in clinical trials, but indicated that they lacked opportunities to do so [18]. Building on these findings by combining insights into reasons for stopping participation with the ability to clearly identify “stayers” who remain continuously involved in research could provide valuable insights and strategies into overcoming barriers affecting investigator participation.

### 4.1 Study limitations

A number of limitations to our study should be noted. First, our results apply only to our specific methods used to define the three clinical investigator phenotypes. However, as discussed, this methodology allowed us to empirically identify the two most important groups of investigators, “stayers” who remain consistently involved in research and “one-and-done” investigators who participate in only a single trial. Second, as noted earlier, the Form FDA 1572 BMIS database does not provide a complete census of clinical investigators and their activities; we also note that definitive information on the number and activity levels of clinical investigators is challenging to ascertain, and estimates vary widely according to survey methods and databases used [16]. Importantly, individual investigators are not required to submit these forms directly to the FDA, as is often assumed to be the case. Although sponsors of clinical trials designed to support regulatory approval must keep a completed 1572 form for each study investigator, they are not required to submit this form to meet the FDA's investigator biographical reporting requirement. Third, while we observed a trend towards greater “one-and-done” investigators in the later years, this could be at least partly explained by some investigators entering the registry for the first time in those later years, and without the opportunity to become a “stop-and-go” or “stayer”. However, we still observed an initial peak of “one and done” in 2000 with a trough in 2007, which could not be attributed to the above limitation, and emphasizes the adverse trends seen in the investigator landscape. Taken together, despite its inherent limitations, the BMIS database remains perhaps the best accessible source for information on investigator activity and participation on FDA-regulated clinical trials. Even assuming a degree of under-reporting, the trends we see for investigator participation are in contrast to current trends in clinical trial activity [23] and ongoing enthusiasm for investment in biopharmaceutical research and development [27].

Finally, since the 1572 BMIS database does not contain information on sub-investigators, it is possible that some “one-and-done” investigators remain active in clinical research but not as primary investigators, which would therefore overestimate the proportion of patients in this category.

## 5 Conclusions

We found that from 1999 to 2015, the number of clinical trial investigators involved in FDA-regulated drug trials who submitted a Form FDA 1572 to the BMIS database declined by approximately one-third. We also found that the proportion of investigators involved in only one trial increased, with a subsequent reduction in investigators who consistently were engaged in clinical research. Further, more of the clinical trial workforce has shifted into predominately non-U.S.-based investigators. These findings, which extend previous work suggesting challenges to the U.S. clinical trials workforce, are potentially concerning. Further research to identify strategies to increase and maintain clinical investigator engagement is needed to help sustain clinical trial activity and bring new therapies to patients.

## Funding statement

This research was funded by the Food and Drug Administration through grant R18FD005292 and cooperative agreement U19FD003800, as well as by pooled membership fees from Clinical Trials Transformation Initiative's member organizations. The views expressed in this publication do not necessarily reflect the official policies of the Department of Health and Human Services.

## Conflicts of interest

Dr. Fordyce has served on advisory boards for Bayer, Novo Nordisk, and Boehringer Ingelheim. Dr. Roe receives research grants from AstraZeneca, Eli Lilly, Janssen Pharmaceuticals, Sanofi-Aventis, Daiichi Sankyo, the Familial Hypercholesterolemia Foundation, and Ferring Pharmaceuticals; consulting fees from Amgen, Astra Zeneca, Bristol Myers Squibb, Eli Lilly, Merck, Daiichi Sankyo, Amgen, Elsevier, Boehringer Ingelheim, PriMed, and Myokardia. None of the other authors has any conflicts of interest to report.
