# Supplementary material for: Trends in clinical trial investigator workforce and turnover: An analysis of the U.S. FDA 1572 BMIS database
Source: Contemp Clin Trials Commun. 2019 May 21;15:100380. doi: 10.1016/j.conctc.2019.100380 (PMC6536616; doi:10.1016/j.conctc.2019.100380)
Supplement: Multimedia component 1 [file mmc1.docx]

**Supplemental Appendix**


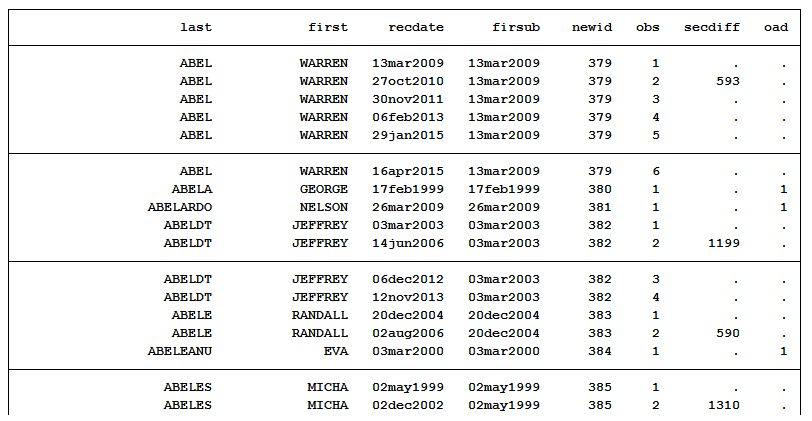


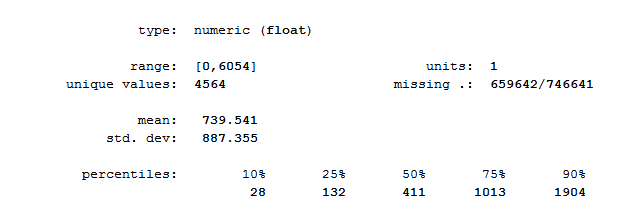


**Supplemental Figure 1. Data extract from BMIS database.**


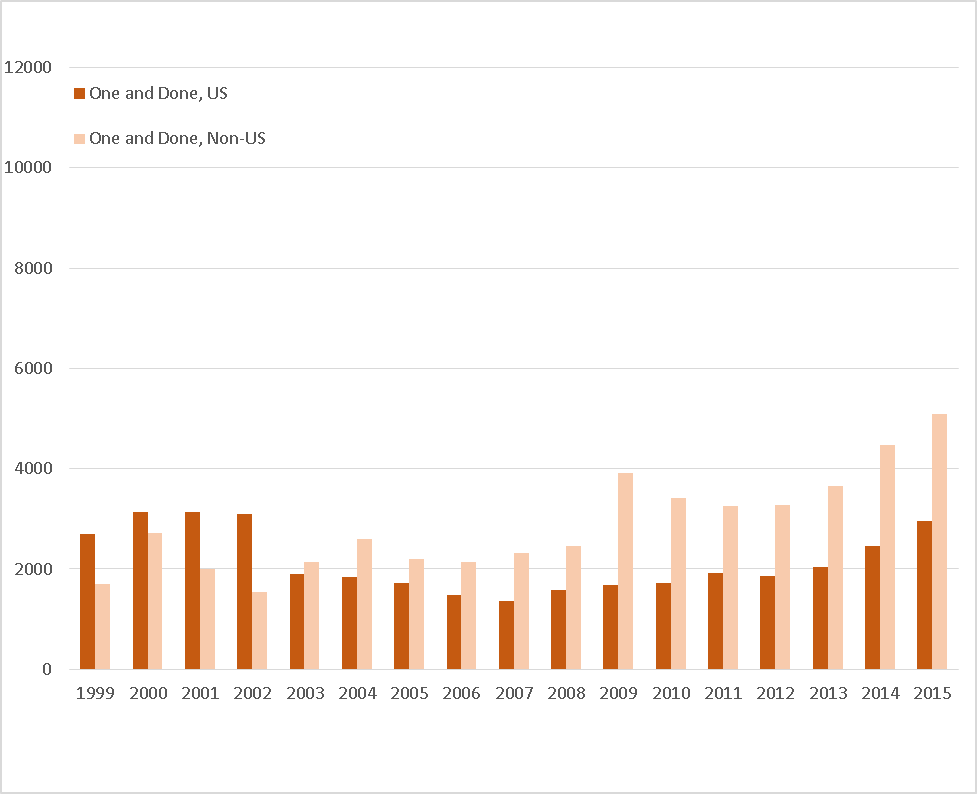


**Supplemental Figure 2a. Temporal trends in investigator turnover according to location: “one-and-done.”**


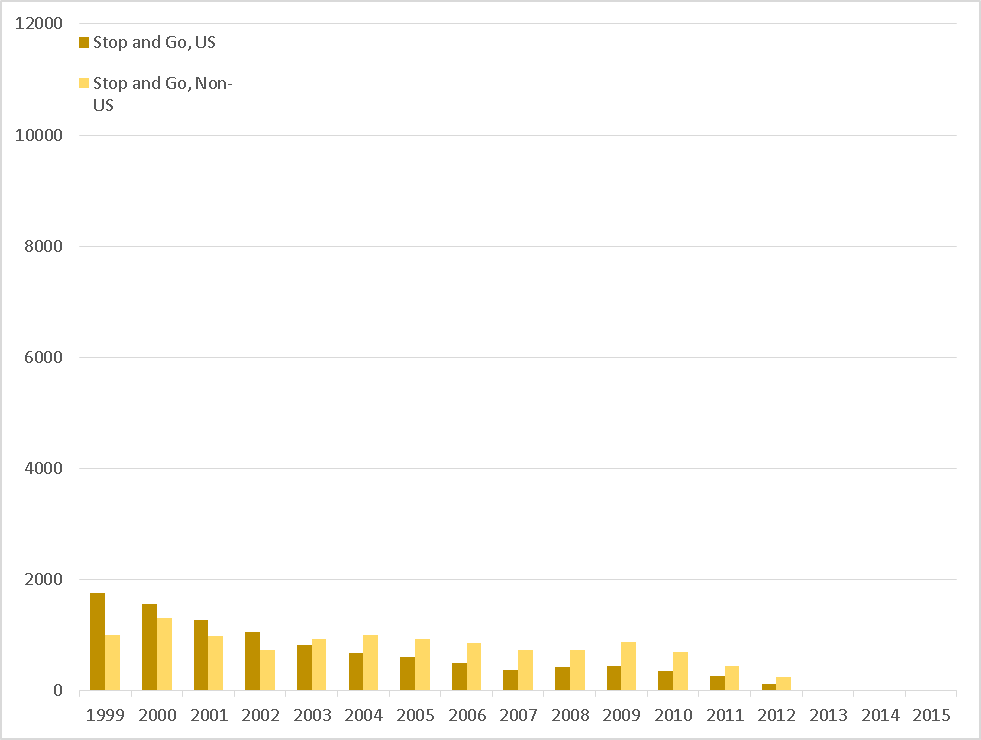


**Supplemental Figure 2b. Temporal trends in investigator turnover according to location: “stop-and-go.”**


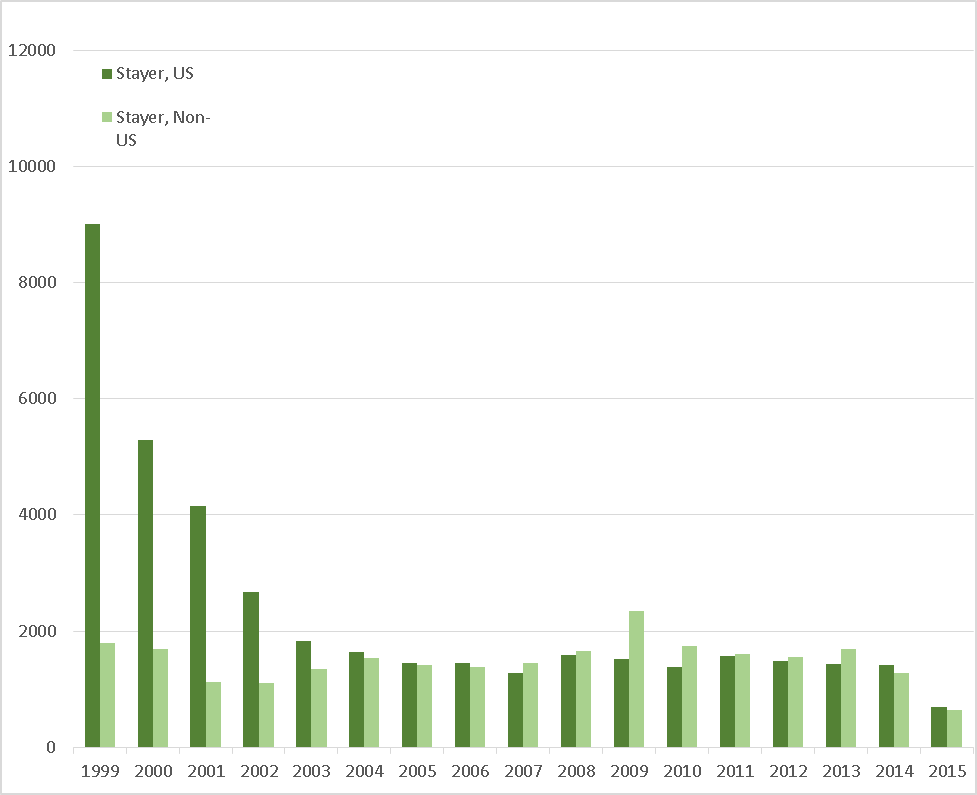


**Supplemental Figure 2c. Temporal trends in investigator turnover according to location: “stayers.”**
